# 

**Published:** 1846-06

**Authors:** 


*Balt. Ed.*

*To Correspondents.*—A. L. H. is referred to the constitution and by-laws of the American Society of Dental Surgeons, for the information he desires in relation to the requirements of applicants for membership to said Society. We would inform him, however, and all others who may be ignorant of the fact, that he must have three members to vouch for his professional abilities and moral character, or submit to an examination before the board of examiners at an annual meeting. . . . We would willingly publish the article of T. P., condemning the use of amalgams for filling teeth, subscribing, as we most heartily do, to the correctness of his views; but as the same arguments and facts have been frequently presented and urged before, we do not think it would be productive of any good. If he desires it, we will return him his manuscript. In the mean time, we would refer him to the Syracuse editor's review of Dr. Allen's letter, which he will find embraces many of the points contained in his communication. . . . Dr. B. is informed that we sent the March number of the Journal to him immediately after it was issued, and as it miscarried, we sent him another, agreeably to his request. . . . N. E. is informed that Mr. Abbey has no agency in Baltimore for the sale of his gold foil. His best plan would be to send his orders direct to Mr. A., 22 Pear-st., Phila. . . . Dr. J. C. D's letter, of April 29th, came to hand at the proper time, and his request was promptly complied with. We shall at all times be pleased to hear from him. . . . J. M. and R. H. are referred to advertisement for the information they desire in relation to the Baltimore College of Dental Surgery. The seventh annual announcement has not yet been issued. As soon as it shall be published, we will send each a copy. The lectures in the Ohio College of Dental Surgery will commence the first week in November; but for further information concerning the School, we would refer our correspondents to Professors Taylor, Cook or Rogers, either of whom, we have no doubt, will take pleasure in furnishing every information which they may desire. . . . In reply to the question of B. L., we state that the practice of separating the front teeth with wedges of soft wood or gum elastic, preparatory to filling cavities in their approximal surfaces, is admissible in young persons, and is, in many cases, preferable to separating with a file; but, in persons more advanced, the practice is often productive of very prejudicial consequences.

*Balt. Ed.*

### INDEX.

PAGE.

A

Art of the Dentist, Complete Elements of the Science and, 331
Address, delivered before Am. Soc. of Dental Surgeons, by E. Parmly, M. D., D. D. S., 3
Address on Professional Excellence, delivered before Am. Soc. of Dental Surgeons, by John Allen, D. D. S., . . 30
Address at the opening of Ohio College of Dental Surgery, by Rev. B. P. Aydelott, D. D., 185
Address, Opening, before Pa. Ass'n of Dental Surgeons, by Robt. Arthur, D. D. S., . 195
American Society of Dental Surgeons, . . . . . 337
American Soc. of Dental Surgeons, sixth annual meeting, 74
American Soc. of Dental Surgeons, Resolutions of, on Use of Amalgams, . . 169, 265
American Soc. of Dental Surgeons, Proceedings of, . 86
American Soc. of Dental Surgeons, seventh annual meeting, . . . . . 337
American Journal and Library of Dental Science, . . 86
American Jour. of Insanity, &c. 259
Arthur, Robt., D. D. S., Opening Address, . . . . 195
Arsenic as a Dental Therapeutic Agent, . . . . . 179
Arsenic as a Remedy for Tooth-Ache, &c. . . . . . 110
Auscultation and Percussion, by M. Barth, . . . 165
Association of Dental Surgeons, Pennsylvania, . . . 208
Aconite, Death from, . . . 255
Abstract of the Med. Sciences, Half-Yearly, . . 71, 259
Anatomical Remembrancer, . 295
Anatomy, Pathological, Elements of, by S. D. Gross, M. D., . . . . . 260
Anatomy, Pathological, by J. Hope, M. D., . . . 258

PAGE.

Arteries and Nerves, Outlines of, by John Neil, M. D., . 260
Allen, C. C., M. D., Letter from, . . . . . . 246
Allen, C. C., M. D., Letter in Reply to, &c., . . . 265
Allen, John, D. D. S., on Professional Excellence, . . 30
Amalgams for filling Teeth, 169, 265
Aydelott, B. P., D. D., Rev., Address of, . . . . 185
Artificial Teeth on Plate with Clasps, . . . 228, 282
Aneurism of the Coronary Artery, . . . . . . 331
Art of the Dentist, Complete Elements of, . . . 331
Artificial Teeth and Palates, Treatise on, . . . 336
Artificial Teeth, on Premature Application of, . . . 282
Addington, R. D., Letter from. 288

B

Bennett, E. S., M. D., Letter from, . . . . . . 60
Buffalo Medical Journal, . 73
British American Journal of Med. and Phys. Sciences, . 73
Baltimore College of Dental Surgery, . . . 163, 261
Baltimore College of Dental Surgery, Museum of, . . 181
Barley Water a Diuretic, . 184
Baker, E., M. D., Dissertation on Diseases of the Gums, 89
Baker, E., M. D., Letter from, 302
Barth, M., on Auscultation and Percussion, . . . . . 165
Brown, B. B., M. D., on Artificial Obturators and Palates, 232

C

City Editor, Washington, . 87
Cavity of a Tooth, Removal of a Drill-head from, . . 87
Correspondents, to, . 88, 181, 339
Correspondence, . . . . 252
Creosote, Effects of, on the Economy in Health, . . 123

PAGE.

Contributions to Operative and Mechanical Dentistry, . 134
College of Dental Surgery, Baltimore, . . 163, 186, 261
College, Pennsylvania, Introductory Lectures in the, . 258
Correction, . . . . . 180
Convention of Dentists, . . 183
Cone, C. O., D. D. S., on the Origin, Development, and Eruption of the Teeth, . 221
Calculi of the Nasal Fossæ, . 254
Calculus, Salivary, . . 338
Claims of the Temperance Reformation, . . . 256
Catalogue, &c., . . . 336

D

Dental Surgeons, American Society of, . . 74, 86, 337
Dental Science, American Journal and Library of, . . 86
Drill-Stock, Improved, . 88
Dental Infirmary, . . . . 88
Dissertation on the Diseases of the Gums, . . . . . 89
Dissertation on Tooth-Ache, 100
Dentistry, Operative and Mechanical, Contributions to, 134
Dentists, on the Education of, 141
Dentists, Convention of, . . 183
Dental Surgery, Baltimore College of, . . . . . 163
Diseases of the Skin, Manual of, 164
Dental College of Ohio, Address before the, . . . . 185
Death from Aconite, . . 255
Dunning, E. J., on Practical Dentistry, . . . . 15
Dissertation on the Elevation of the Dental Profession, . 25
Dissertation on the State of the Dental Profession, . . 38
Dental Intelligencer, . . . 72
Dens Sapientiæ, Necrosis from Pressure of, . . . . . 326
Dentistry, Encyclopedia of, 334
Dentist, Complete Elements of Science and Art of, . . 331
Desirabode, M. M., on Science and Art of the Dentist, . 331
Dentists, Surgeon, in Paris, 338
Deviation in Growth of Tooth, 339
Delinquents, to, . . . . 339

PAGE.

E

Effects of Creosote on the Economy in Health, . . . 123
Evans, G., Letter from, . 132
Elliott, W. H., D. D. S., on Operative and Mechanical Dentistry, . . . . . 134
Education of Dentists, on the, 141
Essay on the Origin, Development and Eruption of the Teeth, . . . . . . 221
Editorship of the Journal, . 263
Evils Resulting from Premature Application of Artificial Teeth, . . . . . . 282
Encyclopedia of Dentistry, . 334
Essay on Trismus Nascentium, 336

F

Fungoid Tumor of Lower Jaw, 330
Filing the Teeth, . . . 146
Filling Teeth, Use of Amalgams for, . . . 169, 265
Forceps, the London, . . 182
Fox, Joseph, M. R. C. S. L., and C. A. Harris, M. D., on Natural History and Diseases of the Teeth, . . . 257
Fungus of Inferior Maxillary, 291
Fungus of Left Maxill'y Sinus, 318

G

Growth and Irregularity of Children's Teeth, . . 69
Gums, Dissertation on the Diseases of, . . . . 89
Great Britain, to our Subscribers in, . . . . 180
Grandhomme, M. A. P., on Regulating the Teeth, . . 334

H

Hare-lip, is the Negro Subject to, . . . . . 58
Half-Yearly Abstract of the Medical Sciences, . 71, 259
Harris, John, M. D., on Removal of Drill-head from the Cavity of a Tooth, . . 87
Harris, John, M. D., on Toothache, . . . . . 100
Harris, John, M. D., on Application of Plates and Obturators, . . . . . 282
Harris, Chapin A., M. D., on

PAGE.
Fungus Tumor of the Antrum, . . . . . 318
Health, Preservation of, . 260
Hope, J., M. D., Principles of Pathological Anatomy, . 258
Harrington, D., on Fungus of Inferior Maxillary, . . 291
Hemorrhagic Diathesis, after Extraction of Molar Tooth, 320

I

Items, General, Suggested by Review of Minutes of Last Meeting A. S. D. S., . . 61
Intelligencer, Dental, . . 72
Infirmary, Dental, . . . 88
Introductory Lectures, . 258
Incorruptible Teeth, . . 264
Indiana Medical College, Catalogue of, &c., . . . 336

J

Journal, British American, of Med. and Phys. Sciences, 73
Journal, Buffalo Medical, . 73
Journal, American, and Library of Dental Science, . . 86
Journal, American, of Insanity, 259
Journal, Southern, of Medicine and Pharmacy, . . . . 259
Journal, Editorship of, . 263
Journal, Medical and Surgical, Illinois, . . . . . 336
Jaw, Lower, Necrosis of, . 324
Jaw, Lower, Fungoid Tumor of, . . . . . . . 330
Jaw Bones, Necrosis of, . 327

L

Letter from E. S. Bennett, M. D. 60
Letter from G. Evans, . 132
Letter from C. C. Allen, M. D., 246
Letter from R. D. Addington, 288
Letter from Syracuse Editor, 310
Letter from E. Baker, M. D., 302
Letter from A. Westcott, M. D., 310
Liston, Robert, Esq., &c., Lectures on Operative Surgery, 258
Lexicon Medical, by David Meredith Reese, A. M., M. D. 72
Lancet, Western, . . 336

M

Minutes of Last Meeting, Review of, . . . 61
Mortimer, W. H., on Children's Teeth, . . . 69

PAGE.
Medicines, their Use, &c., 70
Medical Lexicon, by D. M. Reese, A. M., M. D., . 72
Medical Journal, the Buffalo, 73
Means Employed for Regulating Teeth, . . . 334
Meeting of the American Society of Dental Surgeons, 74
Meeting, Third Annual, of the Va. Soc. of Surgeon Dentists, 167
Manual of the Diseases of the Skin, . . . 164
Manual of Auscultation and Percussion, . . . 165
Midwifery, an Elementary Treatise on, . . 166
Museum of Baltimore College of Dental Surgery, . . 181
Medical Fees, . . . 184
Materia Medica in Rhyme, . 256
Medical Lectures, Introductory, in Med. Dep. of Pa. College, 258
Medical Sciences, Half-Yearly Abstract, . . 71, 259
Medicine and Pharmacy, Southern Journal of, . . 259
Medico-Dental Education, 261
Magnet, Removal of a Drill from Cavity of a Tooth by means of, . . . . 87
Maxillary, Inferior, Fungus of, 291

N

Neligan, J. Moore, M. D., on Medicines, their Use, &c., 170
Nasal Fossæ, Calculi of, . 254
Natural History and Diseases of Human Teeth, . . 257
Neil, John, M. D., on Arteries and Nerves, . . 260
Necrosis of Lower Jaw, . 324
Necrosis from Pressure of the Dens Sapientiæ, . 326
Necrosis of Jaw Bone, . 327

O

Observations on Arsenic as a Remedy for Tooth Ache, 110
On Artificial Teeth on Plate with Clasps, . . 128
On Education of Dentists, 141
On Filing Teeth, . . 146
Opening Address, before Pa. Ass'n of Dental Surgeons, 195
Origin, Development and Eruption of Human Teeth, . 221

PAGE.

Observations on artificial Teeth, Obturators and Palates, . 222
Observations on the Growth and Irregularity of Children's Teeth, . . . 69
Omitted Article, . . . 264
Obituary, . . . 340

P

Parmly, Eleazar, M. D., Address before Am. Soc. D. S., 3
Practical Dentistry, Dissertation on, . . . 15
Profession Dental, Dissertation on the Elevation of the, . 25
Profession, Dental, State of, 38
Professional Excellence, . 30
Proceedings of Am. Soc. D. S., 86
Popular Treatise on the Teeth, 166
Porcelain Teeth, . . 182
Pennsylvania Ass'n of Dental Surgeons, Transactions of, 208
Principles of Path. Anatomy, 258
Preservation of Health, . 260
Pathological Anatomy, Elements of, . . . 260
Premature Application of Artificial Teeth, Palates and Obturators, . . . 282
Pass, Horatio, on Artificial Teeth and Palates, . 336
Pathology and Treatment of Trismus Nascentium, . 336

R

Review of Minutes of Last Meeting Am. Soc. D. S., 61
Ranking, W. H., M. D., Half-Yearly Abstract Med. Sci., 71
Reese, D. M., A. M., M. D., Medical Lexicon, . . 72
Removal of a Drill-head from the Cavity of a Tooth, . 87
Resolutions A. S. D. S. on Use of Amalgams for Filling Teeth, . . 169, 265
Robinson, J., Esq., D. D. S., 184
Robinson, J., Esq., D. D.S., on Filing Teeth, . . 146
Rhyme, Materia Medica in, 256
Rogers, Wm., Ency. of Dent'y, 334

S

Society, Am., of Dental Surgeons, Address before the, 3
Society, Am., of Dental Surgeons, Proceedings of, . 86

PAGE.

Society, Am., of Dental Surgeons, Resolutions of, . 169
Society, Va., of Surgeon Dentists, 3d Annual Meeting of, 167
State of the Dental Profession, Dissertation on, . . 38
Stockton's Dental Intelligencer, 72
Sixth Ann. Meet'g A. S. D. S., 74
Subscribers in Great Britain, 88, 180
Shepherd, S. M., D. D. S., on Artificial Teeth, . . 128
Surgery, Lectures on the Operations of, . . . 258
Surgery, Balt. Col. of Dental, 261
Sims, J. Marion, M. D., on Trismus Nascentium, . 336
Surgeon Dentists in Paris, . 338
Salivary Calculus, . 338

T

Townsand, E., D. D. S., on Elevation of Dental Profession, 25
Taylor, James, D. D. S., on State of Dental Profession, 38
Teeth, Observations on Growth and Irregularities of, . 69
Teeth, Natural History and Diseases of, . . 257
Teeth, Popular Treatise on the, by R. Arthur, D. D. S., . 166
Teeth, Amalgams for Filling, 179
Teeth, Porcelain, . 182, 264
Teeth, Origin, Development and Eruption of the, . 221
Teeth, Means Employed for Regulating the, . . 334
Teeth and Palates, a Treatise on Artificial, . . 336
Temperance Reformation, the, Claims of, . . . 256
Tetanus Successfully Treated, 324
Trismus Nascentium, . 336
Tooth, Dens Sapientiæ, Deviation in Growth of, . 339

V

Velpeau, Alf., A. L. M., M. D., Treatise on Midwifery, . 166
Virginia Society of Dental Surgeons, Trans. of the, 149, 167

W

Westcott, Amos, M. D., on Arsenic as a Remedy for Tooth-Ache, . . . 110
Washington City Editor, . 87

